# A systematic review on deep learning techniques for diabetic retinopathy classification in retinal fundus images

**DOI:** 10.1016/j.isci.2026.116565

**Published:** 2026-06-24

**Authors:** José Araque-Gallardo, Eugenia Arrieta-Rodríguez, Lenis Rueda-Gómez, Oscar Teherán-Forero, Emiro De-La-Hoz-Franco, Margarita Gamarra, Javier Sierra-Carrillo, José Escorcia-Gutierrez

**Affiliations:** 1 Department of Electronic Engineering, Universidad de Sucre, Sincelejo 700001, Colombia; 2 Department of Computational Science and Electronics, Universidad de la Costa, CUC, Barranquilla 080002, Colombia; 3 Department of System Engineering, Universidad del Norte, Puerto Colombia 081007, Colombia; 4 Universidad del Sinú-Elias Bechara Zainúm, Seccional Cartagena, Cartagena 130001, Colombia

**Keywords:** Health sciences, Medical specialty, Medicine, Ophthalmology

## Abstract

Diabetic retinopathy (DR) is the leading cause of preventable blindness worldwide, particularly in low- and middle-income countries. Although expert analysis of color fundus images (CFIs) enables reliable classification, this process is time-consuming. In recent years, deep learning (DL) techniques have demonstrated remarkable performance in analyzing CFI for the detection (binary classification) and multi-class classification of DR. To synthesize recent advances in this field, this study presents a systematic review, analyzing 146 peer-reviewed studies published between 2019 and 2025. This review examines lesion detection and segmentation, as well as DR detection and classification in CFI using two approaches: direct full-image and lesion-based classification. In addition, a brief scientometric analysis was performed to identify publication trends, leading journals, and countries contributing to this domain. The insights provided by this review aim to support researchers in selecting effective strategies and advancing the development of DL-based systems for the detection and classification of DR.

## Highlights

• Reviews deep learning methods for diabetic retinopathy analysis in fundus images
• Compares lesion-level and image-level deep learning paradigms
• Discusses strengths and limitations of current deep learning approaches
• Explores explainability and emerging paradigms for diabetic retinopathy screening

## Graphical abstract

**Figure:**
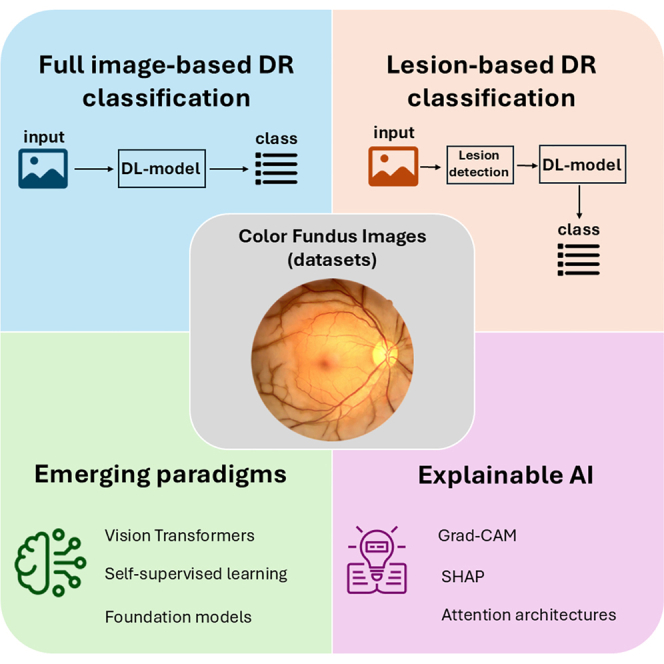

## Introduction

**Figure 1:**
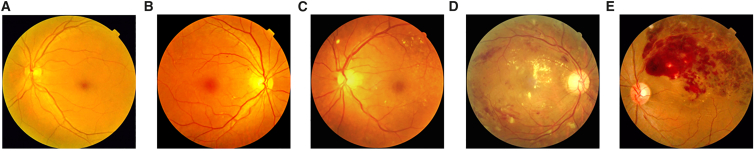
Representative color fundus images illustrate the progression of diabetic retinopathy (DR) severity (A) Healthy. (B) Mild NPDR. (C) Moderate NPDR. (D) Severe NPDR. (E) PDR with neovascularization.

The World Health Organization (WHO) reports that diabetes mellitus (DM) is one of the leading causes of blindness, kidney failure, heart attack, stroke, and lower-limb amputation.^1^ The International Diabetes Federation (IDF), in its 2021 Disease Atlas, estimated that 537 million adults worldwide live with DM, with a standardized prevalence of 10.5%, mostly in low- and middle-income countries. The IDF also highlighted that if the trend continues, 783 million adults will suffer from DM by 2045.^2^ In the Americas region, according to estimates from the Pan American Health Organization, 62 million people lived with diabetes in 2022.^3^ DM directly causes diabetic retinopathy (DR), a complication in which high blood glucose levels block blood vessels in the eye, leading to swelling and leakage of blood or fluid, which can damage the eye. The International Agency for the Prevention of Blindness estimates that one in three individuals with diabetes has some level of DR, and one in ten develops a form of vision-threatening disease. By 2020, this has led to 1 million people worldwide going blind due to DR, while nearly 3 million will have moderate to severe visual impairment from the condition,^4^ which can be diagnosed and classified based on alterations in the retina caused by DM. It is challenging to detect morphological changes in color fundus image (CFI), such as microaneurysms (MAs), hard exudates (HEs), soft exudates (SEs), hemorrhages (HMs), and neovascularization (NV), as well as changes in the optic disc, vessels, and macula. Computer-aided diagnosis (CAD) systems can help identify these changes and assist ophthalmologists in diagnosing the condition. DR follows a natural progression through various levels of severity, allowing it to be classified into five levels based on lesions and observable pathologies, as shown in Figure 1 and Table 1.^5^

**Table 1:** Diabetic retinopathy severity levels

DR severity	Morphological characteristics
No DR (healthy)	without abnormalities
Mild non-proliferative retinopathy	only MA
Moderate non-proliferative retinopathy (NPDR)	MA and at least one of the following: HEs, SEs, and EX.
Severe proliferative retinopathy	any of the below: prominent intraretinal microvascular abnormalities. definite venous beading in two quadrants. NO sign of PDR.
Proliferative retinopathy (PDR)	NV or vitreous/pre-retinal hemorrhage

Early detection and classification of DR is essential for preventing vision loss and enhancing the quality of life of patients. It also reduces treatment costs, and supports better management of DR. Recent advances in deep learning (DL) have significantly impacted several medical imaging applications, including retinal disease analysis, skin lesion classification, and automated diagnostic support systems.^6^ Therefore, the demand for effective healthcare solutions, such as DL-based CAD systems for the early diagnosis of DR, is increasing. This study aimed to review the current literature on DL for detecting lesions and classifying DR in CFIs. By analyzing trends, challenges, and opportunities in this field, we aim to contribute to the development of more accurate and efficient diagnostic tools to improve the clinical outcomes of patients with DR.

This review provides up-to-date information for researchers and medical personnel developing DL models for detecting and classifying DR. It synthesizes traditional lesion detection methodologies, emerging paradigms, and the critical role of explainability in clinical decision making. In addition to this technical synthesis, a scientometric analysis offers a comprehensive overview of the research trends and institutional contributions. Furthermore, this review provides an extensive list of publicly available datasets to help investigators identify suitable data for their experiments.

## Results

### Included studies

This systematic review was conducted in accordance with the preferred reporting items for systematic reviews and meta-analyses (PRISMA) 2020 guidelines to ensure transparency and reproducibility of the study selection process. A comprehensive search was conducted in Scopus, Web of Science, and IEEE Xplore, covering studies published from 2019 to 2025. This review focused on research addressing lesion detection, segmentation, and DR detection or classification using CFIs. CFIs remains the most widely available and commonly used imaging modality in large-scale DR screening.

**Figure 2:**
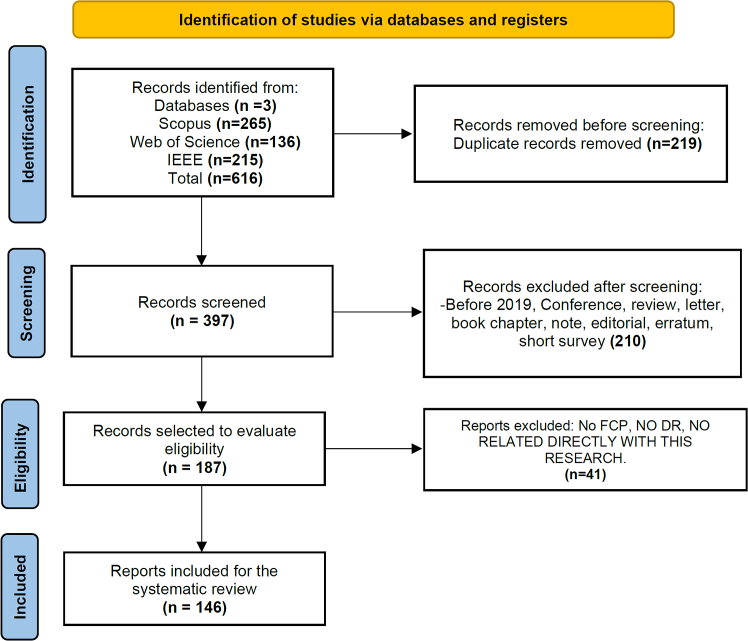
PRISMA flow diagram of the literature selection process summarizing the identification, screening, eligibility assessment, and inclusion of studies identified through the search strategy

Eligible studies included peer-reviewed English-language journal articles that used non-DL or DL approaches for lesion-level analysis or DL methods for detecting and classifying DR. Studies that used alternative imaging modalities or were not directly related to DR analyses were excluded. A total of 616 records were initially identified, and after duplicate removal and screening, 146 studies were included in the final qualitative synthesis. Figure 2 summarizes the PRISMA flowchart of this study.

Further details regarding the search strategy, eligibility criteria, study selection process, and data extraction are provided in the STAR Methods section.

### A brief scientometric analysis

**Figure 3:**
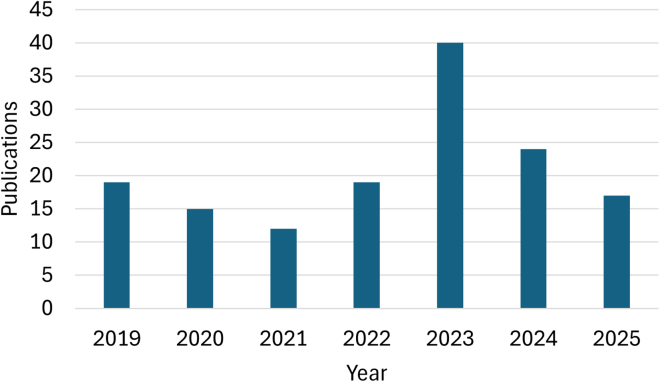
Distribution of studies included in this review by publication year, illustrating research activity trends in deep learning for diabetic retinopathy analysis

**Figure 4:**
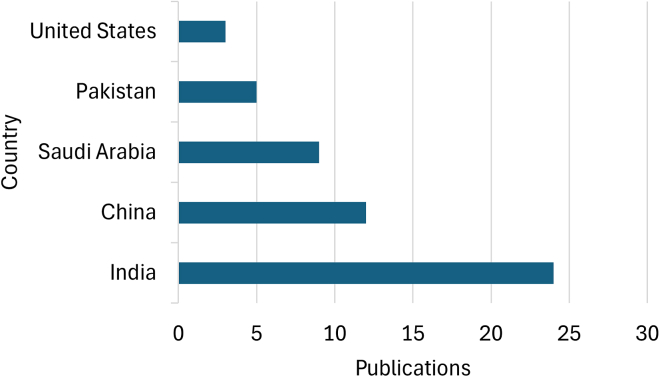
Top five countries contributing to the studies included in this review

**Figure 5:**
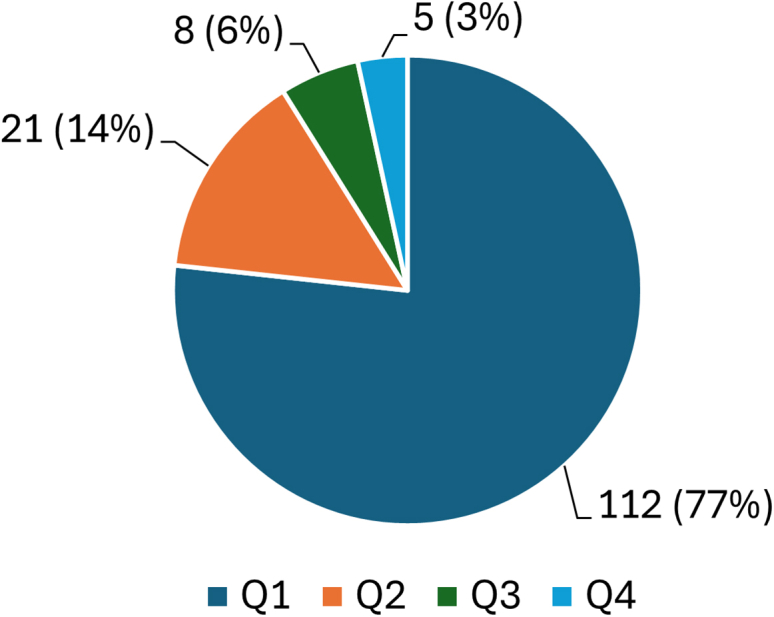
Number of reviewed studies published in journals, classified by quartile ranking

**Figure 6:**
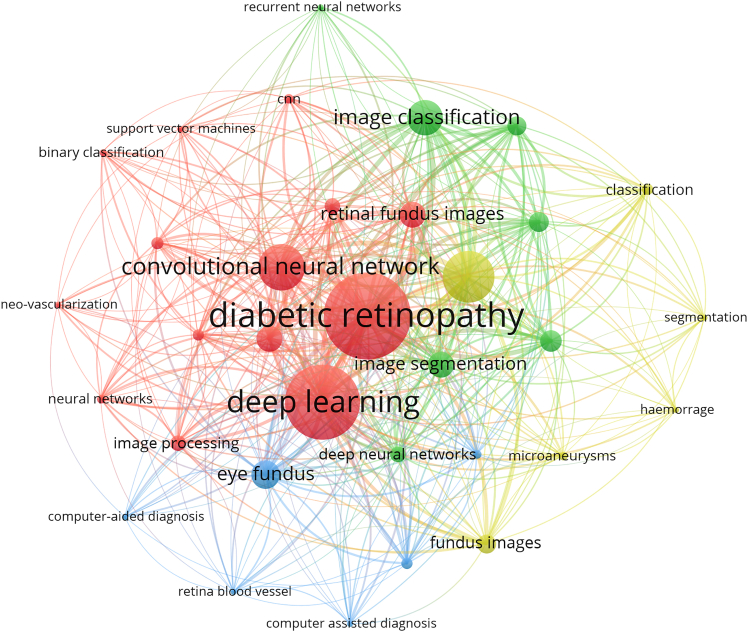
A keyword co-occurrence network illustrating relationships and primary research topics in the reviewed literature

A brief scientometric analysis was conducted to characterize the research landscape of DL applications for DR. The analyzed variables included publication year, country of origin, journals, and keyword co-occurrence networks (Figures 3, 4, 5, and 6, and Table 2).

**Table 2:** Top-ranked journals for publications

Ranking	Journal	ISSN	Country	Quartile (SJR)	Publications
1	IEEE Access	2169–3536	United States	Q1	15
2	multimedia tools and applications	1380–7501	Netherlands	Q1	15
3	biomedical signal processing and control	1746–8094	Netherlands	Q1	11
4	IEEE transactions on medical imaging	0278–0062	United States	Q1	5
5	sensors	1424–8220	Switzerland	Q1	4

Temporal analysis revealed sustained growth in research activity on DL-based approaches for DR detection and classification using CFIs. As shown in Figure 3, the number of publications increased from 19 in 2019 to a peak of 40 in 2023, despite a temporary decline in 2021 and a moderate decrease in 2024–2025. Overall, this trend reflects the rapid consolidation of DL, particularly convolutional neural networks (CNNs), as the dominant paradigm in CAD systems for ophthalmology. This growth is closely associated with the increasing availability of large, annotated datasets, which have enabled more robust model development and evaluation.

From a geographic perspective, the distribution of publications is concentrated in a limited number of countries. As illustrated in Figure 4, India leads with 24 publications, followed by China (12), Saudi Arabia (9), Pakistan (5), and the United States (3), collectively accounting for approximately 36% of the analyzed corpus. This concentration suggests that DL-based DR research is strongly driven by emerging economies, particularly in Asia, where there is a high prevalence of diabetes and increasing investment in artificial intelligence (AI) research. However, the remaining 64% of studies were distributed across a wide range of countries, indicating a progressively global research effort and highlighting opportunities for broader international collaboration.

The analysis of publication venues (Figure 5 and Table 2) indicates that most studies are published in high-impact journals. IEEE Access and multimedia tools and applications (both Q1) led with 15 publications each, followed by biomedical signal processing and control (Q1) with 11 publications. Notably, 76.7% of the studies were published in Q1 journals and 14.4% in Q2 journals, meaning that over 91% of the literature originated from top-tier venues. This distribution reflects the strong methodological maturity and technical rigor of current research in DL-based DR analysis, as well as the high level of interdisciplinary integration between AI and medical imaging techniques.

The keyword co-occurrence network (Figure 6) further highlights the field’s structural organization. The terms “diabetic retinopathy” and “deep learning” emerged as central hubs connecting key research directions, including image segmentation, classification, and fundus image analysis. Distinct clusters reflect specialized subdomains: One cluster emphasizes DL-based diagnostic modeling (e.g., neural networks, feature extraction), another focuses on clinical detection and classification tasks, and a third addresses low-level image processing and vessel analysis. Additionally, the presence of pathology-specific terms, such as HE and NV, indicates a growing interest in lesion-level analysis, reinforcing the relevance of combining image- and lesion-based approaches within the DR research landscape.

### Datasets and metrics

Multiple publicly accessible CFI datasets are now available, facilitating the implementation of computer algorithms for the analysis of DR and its associated lesions. These datasets mainly focus on specific regions of the retina or symptoms of DR. Researchers have explored these datasets for various goals, including lesion detection and DR classification; therefore, choosing the right dataset for evaluating the performance of the developed algorithms should help ensure accurate disease diagnosis. A summary of the most relevant datasets for DR, along with their main characteristics, is presented in Table 3.

**Table 3:** Publicly available retinal fundus image datasets

N°	Dataset	Number of images	DR classes	Lesion masks	Image resolution
1	MESSIDOR	1200	4 (grades 0–3)	no	1440 × 960, 2240 × 1488, 2304 × 1536
2	MESSIDOR-2	1748	4 (grades 0–3)	no	1440 × 960, 2240 × 1488, 2304 × 1536
3	EYEPACS (Kaggle)	88702	5 (grades 0–4)	no	–
4	DIARETDB0	130	2 (normal/abnormal)	yes	1500 × 1152
	DIARETDB1	173	2 (normal/abnormal)	yes	1500 × 1152
5	DriDB	50	3 (NoDR/NPDR/PDR)	yes	720 × 576
6	DRIONS-DB	110	not applicable	yes (optic disc only)	600 × 400
7	DRIVE	40	not applicable	yes (vessel segmentation)	768 × 584
8	STARE	400	not applicable	yes (vessel segmentation)	605 × 700
9	e-ophtha	463	not applicable (lesion-specific)	yes (MA, exudates)	1440 × 960, 2544 × 1696
10	CHASE-DB1	28	not applicable	yes (vessel segmentation)	1280 × 960
11	FGADR	1842	5 (grades 0–4)	yes (MA, EX, SE, HE, NV)	2136 × 3216
12	HEI-MED	169	not applicable	yes (MA, exudates)	2196 × 1958
13	Review	16	not applicable	yes (MAs, exudates, HE)	3584 × 2438, 1360 × 1024, 2160 × 1440
14	DRISHTI-GS	101	not applicable	yes (optic disc and cup)	2047 × 1760
15	ARIA	212	3 (normal/NPDR/PDR)	no	768 × 576
16	ViCAVR	58	2 (normal/abnormal)	yes (vessels, lesions)	768 × 584
17	ROC (MAs)	100	not applicable	yes (MA)	768 × 576, 1058 × 1061, 1389 × 1383
18	HRF	66	not applicable	yes (vessels, optic disc)	3504 × 2336
19	ONHSD	99	not applicable	yes (optic nerve head)	640 × 480
20	DR HAGIS	39	not applicable	yes (vessels, optic disc, lesions)	4752 × 3168, 2816 × 1880, 3456 × 2304, 2896 × 1944, 3216 × 2136
21	IDRID	516	5 (grades 0–4)	yes (vessels, optic disc, lesions)	4288 × 2848
22	FIRE	134	not applicable	yes (vessels)	2912 × 2912
23	RODREP	1120	4 (grades 0–3)	yes (vessels, exudates, HE)	2000 × 1312

This review reports on the performance of standard evaluation metrics commonly employed in DR analysis. For detection and classification tasks, performance is typically assessed using accuracy (Acc), sensitivity (Se, also referred to as recall), specificity (Sp), precision (Pr), and F1-score, all of which are derived from the confusion matrix, which quantifies true positives (TPs), true negatives (TNs), false positives (FPs), and false negatives (FNs). In addition, threshold-independent metrics, such as the receiver operating characteristic (ROC) curve and its corresponding area under the curve (AUC), are frequently used to evaluate the model discrimination capability.

For lesion detection and segmentation tasks, overlap-based metrics, such as the Dice coefficient (Dice) and intersection over union (IoU), are widely used to quantify the agreement between predicted masks and ground-truth annotations, particularly in the presence of small and sparse lesions. In detection settings, metrics such as mean average precision (mAP) are employed to evaluate the localization performance.

Notably, the choice of metric can significantly influence the interpretation of performance, especially in imbalanced datasets, which are common in DR analysis. Therefore, multiple complementary metrics are typically reported to provide a more comprehensive evaluation of the model’s performance.

Furthermore, class imbalance in many DR datasets can undermine the reliability of commonly reported metrics, such as Acc, necessitating the use of complementary measures, such as Se, Sp, and AUC. In addition, differences in annotation quality and the absence of standardized evaluation protocols further complicate benchmarking efforts, highlighting the need for more consistent evaluation frameworks in this area.

### Lesion detection in CFIs

**Figure 7:**
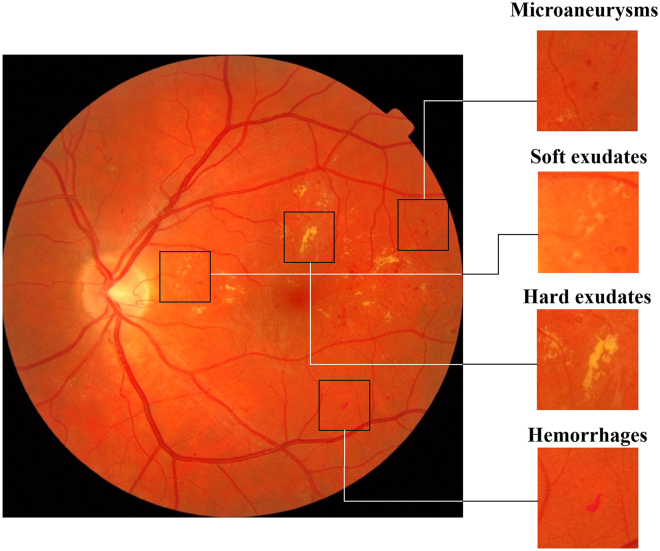
Representative examples of lesions commonly analyzed for diabetic retinopathy detection and classification in color fundus images

This section reviews the methods for detecting retinal lesions in color CFIs, which are key biomarkers for the diagnosis and progression of DR. These lesions include MAs, HEs, SEs, EXs, and NV, each reflecting different pathological processes, such as vascular leakage, ischemia, or abnormal vessel growth. Figure 7 illustrates the visual characteristics of these lesions.

Existing approaches for lesion detection in CFIs can be broadly categorized into non-DL and DL methods, respectively. Traditional approaches rely on handcrafted feature extraction, combined with image processing techniques such as thresholding, morphological operations, and region-based segmentation, followed by classification with conventional machine learning algorithms. Although these methods have been effective in controlled settings, their performance is often dependent on carefully designed preprocessing and feature engineering, limiting their robustness across heterogeneous datasets.

**Figure 8:**
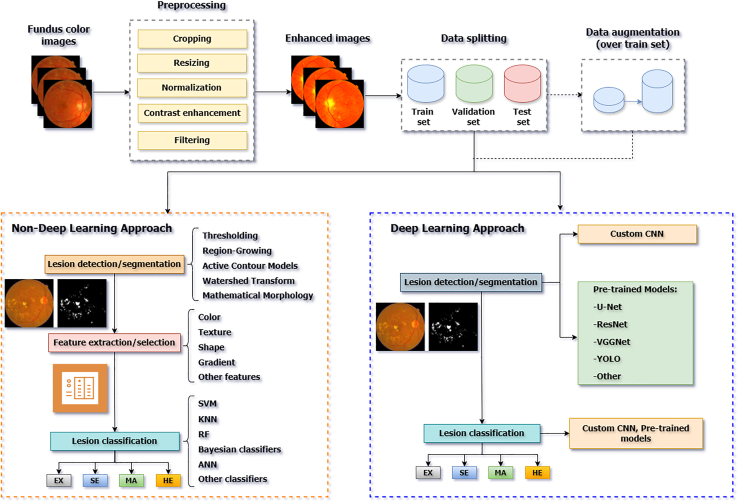
General framework for lesion detection in color fundus images

In contrast, DL-based approaches leverage CNNs and related architectures to learn hierarchical feature representations directly from data, thereby enabling end-to-end detection and segmentation of lesions. These methods have demonstrated improved performance and adaptability, particularly when trained on sufficiently large and well-annotated datasets. Figure 8 presents a general framework for lesion detection in CFIs.

#### Non-DL approach for lesion detection

This section presents a comprehensive overview of non-DL approaches for retinal lesion detection in CFIs. These methods primarily rely on handcrafted feature extraction and traditional image processing techniques, including morphological operations, intensity transformations, filtering, and clustering, and are combined with classical machine learning classifiers such as k-nearest neighbors (KNNs), support vector machines (SVMs), and random forests. Although these approaches have been widely explored for detecting different lesion types, including EXs, SEs, MAs, HEs, and NV, their performance often depends on carefully designed features and preprocessing strategies. The following subsections describe the methodologies developed for each lesion type, highlighting their key characteristics and the processing pipelines.

Traditional non-DL approaches for EXs and SEs in CFIs primarily rely on intensity-based analysis and handcrafted features, leveraging the high reflectance of exudates to facilitate their identification. Early methods employed green-channel extraction and histogram-based thresholding to isolate candidate regions,^7^ while clustering and transformation-based techniques, such as fuzzy C-means, entropy filtering, histogram equalization, and discrete wavelet transform, were used to enhance lesion contrast and support classification using conventional models, including KNN, SVM, and artificial neural networks (ANN).^8^^,^^9^

To improve segmentation Acc, several approaches have incorporated morphological processing and edge-based refinement strategies. These include combinations of fuzzy clustering with edge detection and adaptive thresholding,^10^ as well as morphological operations integrated with fuzzy logic-based classification.^11^ Additional pipelines have employed image enhancement techniques, including CLAHE and region of interest localization, to support lesion quantification under clinically relevant criteria.^12^

More advanced methods have focused on feature optimization and region-based representations. These include the integration of dimensionality reduction and optimization algorithms^13^ and feature extraction using autoencoders and handcrafted descriptors.^14^ Superpixel-based segmentation combined with pixel-level classification has also been explored to improve spatial coherence in lesion detection.^15^ In contrast, hybrid approaches integrating multiple classifiers, including MLP, SVM, H-ANFIS, and CNN, have been proposed to enhance exudate detection.^16^

Overall, while these methods demonstrate effectiveness in controlled settings, their performance remains highly dependent on preprocessing quality and handcrafted feature design, limiting their robustness and generalization to heterogeneous datasets.

MA detection in CFIs is particularly challenging because of the small size and low contrast of these lesions, which has led most approaches to adopt a candidate extraction followed by a classification strategy. Early work explored peak detection mechanisms combined with genetic programming for classification.^17^

Feature-based representations have been widely investigated, including the use of gray-level co-occurrence matrix (GLCM) descriptors and multi-scale features derived from green and grayscale channels, typically combined with classifiers such as KNN and MLP.^18^^,^^19^

In parallel, intensity-driven and transformation-based techniques have been employed to enhance candidate detection, including pixel intensity rank transform (PIRT), non-local mean filtering, and local Fourier transform (LFT)-based feature enhancement, followed by classification using SVM, random forest, or neighborhood-based approaches.^20^^,^^21^^,^^22^^,^^23^

Efforts to improve the discrimination between lesions and vascular structures have incorporated feature fusion and refined candidate extraction strategies, such as local cross-section transformations, sliding-band filtering, and ensemble-based classification.^24^^,^^25^ Texture descriptors, particularly local binary patterns (LBPs), have also been extensively used, with extensions based on multi-kernel SVM and hybrid feature representations.^26^^,^^27^

Finally, morphology-driven pipelines have been applied to enhance lesion localization by integrating shape-constrained features, region-context modeling, and connected component analysis for improved candidate refinement.^28^^,^^29^

Despite these developments, MA detection remains a critical bottleneck because its performance is highly sensitive to image quality and lesion visibility, often resulting in reduced Se and limited generalization across heterogeneous datasets.

Compared with other lesion types, HEs and NV have received relatively limited attention in non-DL approaches, which reflects both their lower representation in the available datasets and the increased difficulty associated with their detection.

Existing HE methods primarily rely on visual feature extraction and region-based segmentation strategies. For example, approaches combining image enhancement, 2D Gaussian modeling, and watershed segmentation have been used to capture lesion appearance characteristics,^30^ while alternative frameworks have explored window-based feature extraction followed by SVM classification to refine candidate regions.^31^

In contrast, NV detection has been addressed through vessel-centric analysis, in which the enhancement of vascular structures is used in multi-scale abnormal growth patterns. Techniques based on morphological filtering, including bottom-hat transformation and green-channel processing, combined with thresholding and clustering, have been proposed to differentiate pathological vessels from normal vasculature.^32^

The underrepresentation of HE and NV in existing datasets directly impacts the development of reliable detection methods. Variations in lesion appearance further complicate modeling, highlighting the importance of more comprehensive annotations and standardized evaluation.

Approaches for the simultaneous detection of multiple retinal lesions have focused on structured feature representations and multi-stage pipelines that integrate candidate extraction and classification. Superpixel-based frameworks have been used to capture the local context and support the joint detection of red lesions, such as HEs and MAs.^33^ Methods based on geometric patterns and hierarchical segmentation have incorporated sequential learning strategies, including recurrent neural networks, to model the spatial relationships between lesion types,^34^ while hybrid pipelines combining morphological preprocessing, dimensionality reduction, and clustering have been applied to improve early stage lesion detection.^35^

Alternative formulations have explored image decomposition and statistical modeling to better represent lesion variability. Layer-based decomposition strategies have been proposed to isolate candidate regions prior to classification,^36^ whereas anomaly detection frameworks based on probabilistic modeling have been used to distinguish lesions from background structures using patch-based representations and statistical regularization.^37^

Although these approaches enable the joint detection of multiple lesion types, their reliance on heterogeneous feature representations and sequential processing limits their ability to consistently capture the interactions between lesions. In addition, variability in lesion co-occurrence patterns and the lack of standardized multi-lesion benchmarks further complicate performance evaluation and comparison. These limitations highlight the inherent difficulty in modeling multiple lesions within traditional frameworks and motivate the adoption of more integrated approaches in subsequent developments.

#### Deep learning approach for lesion detection

DL approaches have improved retinal lesion detection in CFIs by enabling end-to-end learning of discriminative feature representations. In contrast to traditional methods that depend on handcrafted features and sequential processing steps, DL models, particularly CNNs and their variants, integrate feature extraction, representation learning, and prediction within unified frameworks. These approaches have been applied to the detection and segmentation of different DR lesions, including exudates, MAs, HEs, and NV, as well as multi-lesion analysis. The following subsections summarize the main DL-based strategies, emphasizing architectural design, preprocessing schemes, and methodological trends.

EX detection has evolved from detection-oriented frameworks to segmentation-driven architectures, with an increasing emphasis on multi-scale feature representation and attention mechanisms. Early detection strategies combine object detection models and downstream classification. For example, hybrid pipelines integrating YOLO-based candidate detection with lightweight CNN classifiers have been proposed to improve efficiency and data availability through synthetic augmentation.^38^

Recent studies have focused on enhancing feature representation using fusion and attention mechanisms. Architectures incorporating multi-source inputs, such as green channels, morphological features, and contrast-enhanced images, combined with attention modules, have been shown to improve the discrimination of EX regions under varying imaging conditions.^39^

Encoder-decoder architectures based on U-Net remain the dominant paradigm for EX segmentation. Variants incorporating residual, inception, and multi-scale feature fusion modules, often combined with channel attention, have been widely used to improve the detection of small and sparse lesions.^40^^,^^41^ Additional strategies have addressed the lesion size imbalance through specialized network designs and loss functions, enabling more balanced learning across lesion scales.^42^

Hybrid and multi-stage frameworks have further explored refinement strategies by combining initial segmentation with mask-guided enhancement or integrating classical preprocessing with deep models.^43^^,^^44^ Generative approaches based on adversarial learning have also been investigated to enhance feature representation, particularly through frequency-domain processing and tailored loss formulations.^45^

Overall, DL-based methods have significantly improved EX detection performance by enabling end-to-end feature learning and robust representation of lesion variability. However, their effectiveness is influenced by dataset characteristics and lesion distribution, particularly in the presence of small or sparsely distributed exudates. Table 4 summarizes the studies on exudate detection using DL techniques.

**Table 4:** Summary of exudate detection using DL techniques

Authors	Preprocessing	Methods	Dataset	Performance
Hussain et al.^38^	N/A	YOLOv5M custom CNN classifier	EYEPACS	Pr = 99% Se = 100% F1-score = 100%
Guo et al.^39^	green channel extraction, CLAHE, gaussian curvature	YOLOv3, RAM, CNN	MESSIDOR HEI-MED E-Ophtha EX	Acc = 93% Acc = 94% Acc = 92%
Zong et al.^40^	green channel extraction, CLAHE, gamma correction	SLIC superpixel segmentation, U-Net	IDRiD	Se = 96.38% Sp = 97.14% Acc = 97.95%
Do et al.^43^	image patching	VGGunet HRNetv2 + OCR	IDRiD DDR Ehos	AUPR = 89.30%, mDSC = 76.60%, mIoU = 62.40% AUPR = 69.30%, mDSC = 60.80%, mIoU = 45.50% AUPR = 68.90%, mDSC = N/A, mIoU = N/A
Fu et al.^41^	image contrast enhancement, horizontal flip, up-down flip, and random rotation.	RMCA-Unet, MSFF	IDRiD Kaggle local dataset	Acc = 99.47%, F1score = 79.65%, AUC = 98.63% Acc = 99.63%, F1score = 80.99%, AUC = 99.19% Acc = 99.89%, F1score = 54.43%, AUC = 98.86%
Shujaat et al.^44^	resizing, noise removal, channel extraction	AlexNet CNN Classifier	local dataset DIARETDB1	Se = 100%, Sp = 93.75%, Acc = 96.60% Se = 98.80%, Sp = 98.78%, Acc = 98.88%
Liu et al.^42^	data augmentation (resizing, flipping, rotating)	Dual-branch CNN	DDR IDRiD	IoU = 40.47%, F1-score = 57.62%, AUPR = 50.68% IoU = 61.19%, F1-score = 75.92%, AUPR = 77.67%
Eftekhari et al.^46^	color normalization, eliminate retina background, median filtering	CNN (segm.) CNN classifier	ROC E-Ophtha-MA	Se = 76.90% Se = 77.10%
Kar et al.^45^	green channel enhancement, wavelet decomposition	GAN MSR-net	Idrid E-Ophtha	F1-score = 82.46% F1-score = 84%

The detection of MAs using DL remains challenging owing to their small size and low contrast, which has driven the development of architectures focused on multi-stage refinement and enhanced feature representation. Early CNN-based frameworks adopted sequential pipelines in which candidate regions extracted from probability maps were further refined through pixel-level classification,^46^ while alternative approaches combined preprocessing, vessel suppression, and patch-based CNN models to improve discrimination between lesions and background structures.^47^

Subsequent developments have emphasized multi-scale and hierarchical feature-learning to preserve the fine-grained lesion information. Architectures based on pyramid representations and joint segmentation-classification strategies have been proposed to better capture lesion variability across scales.^48^^,^^49^ Complementarily, attention mechanisms have been incorporated to enhance feature fusion and exploit spatial relationships between MAs and vascular structures, improving localization Acc.^50^

Encoder-decoder models, particularly U-Net and its variants, remain central to MA segmentation. These approaches often integrate false-positive reduction stages, specialized loss functions, or modified skip connections to address class imbalances and complex lesion patterns.^51^^,^^52^ Ensemble strategies that combine multiple architectures have also been explored to improve robustness and segmentation performance.^53^

Further improvements have been pursued through hybrid and optimization-based strategies, integrating CNN architectures with feature descriptors, region-based segmentation, or autoencoder frameworks to enhance the detection performance.^54^^,^^55^ Attention-enhanced models have also been proposed to refine feature representation under imbalanced conditions.^56^

Performance remains constrained by the intrinsic characteristics of MAs, as their subtle appearance continues to challenge detection Se, even in DL frameworks, particularly under variations in image quality and dataset composition. Table 5 presents an overview of these studies.

**Table 5:** Summary of microaneurysm detection using deep learning techniques

Authors	Preprocessing	Methods	Dataset	Performance
Zhang et al.^48^	laplacian local attention patching	T-net	ROC DIARETDB1 E-Ophtha retina check IDRID	Se = 83.30%, Pr = 99.90%, Sp = 99.90%, F1-score = 90.90% Se = 80.30%, Pr = 99.90%, Sp = 99.90%, F1-score = 89.10% Se = 96.00%, Pr = 85.20%, Sp = 99.90%, F1-score = 90.30% Se = 99.80%, Pr = 87.00%, Sp = 99.90%, F1-score = 93.00% Se = 99.90%, Pr = 44.00%, Sp = 99.80%, F1-score = 61.10%
Zhang et al.^50^	green channel extraction, CLAHE, Otsu threshold	Multilevel attention mechanism (CNN)	IDRID	Pr = 89.50% Se = 86.80%
Astorga et al.^51^	gamma correction, CLAHE	U-Net ResNet classifier	IDRID E-Ophtha	AUPR = 52.54% AUPR = 54.11%
Bindhya et al.^54^	adaptive wiener filter	bayesian U-Net, morphological top-hat transform, DCNN classifier	IDRID	Acc = 91.70% Se = 91.80% Sp = 91.20%
Xia et al.^49^	N/A	MSRNet MS-efficient net classifier	E-ophtha-MA IDRID RC-RGB-MA	F1-score = 71.50%, DSC = 99.70%, AUC-PR = 61.50%, AUC-ROC = 99.80% F1-score = 99.90%, DSC = 99.90%, AUC-PR = 82.40%, AUC-ROC = 99.90% F1-score = 99.50%, DSC = 99.50%, AUC-PR = 77.90%, AUC-ROC = 99.50%
Liao et al.^52^	grayscale transformation, patch extraction	One-stage encoder-decoder network with skip subtraction fusion	E-ophtha-MA ROC	Se = 78.19% Se = 55.90%
Raudonis et al.^53^	cropping regions of interest (ROIs) using an overlapping sliding window	Ensemble of U-Net, ResNet34-UNet, and UNet++	custom dataset	IoU = 91% DSC = 95%
Abbas^55^	N/A	CRGA algorithm R-CNN classifier	a mix of selected images from ROC, DIARETDB1, and MESSIDOR	Se = 92% Sp = 95% TPR = 93% FPR = 65% AUC = 94%
Vanaja and Prakasam^56^	green channel extraction, resizing, CLAHE, and denoising	CBAM-AG U-net	IDRID	Dice = 86.5% AUC = 99.6%

DL-based approaches for HE and NV detection remain relatively limited compared to other lesion types, reflecting both dataset constraints and the complexity of modeling these pathologies. Existing methods primarily adopt segmentation and classification frameworks based on CNNs. For HE detection, modified U-Net architectures combined with preprocessing steps such as green-channel extraction, normalization, and data augmentation have been used to improve lesion delineation,^57^ while alternative pipelines have incorporated region-based segmentation and sequence modeling strategies to refine classification performance.^58^

In the case of NV, approaches have focused on enhancing vascular structures and leveraging CNN-based segmentation and classification models. Techniques based on contrast enhancement, patch-based training, and semantic segmentation have been proposed to improve the detection of abnormal vessel growth,^59^ along with transfer learning strategies using pre-trained architectures such as ResNet and GoogleNet to support classification tasks.^60^ Tables 6 and 7 provide the representative works for HE and NV detection using DL techniques.

**Table 6:** Summary of hemorrhage detection using DL techniques

Autors	Preprocessing	Methods	Dataset	Performance
Skouta et al.^57^	removal of the black border, extract the green channel, data augmentation	Unet	a mix of IDRiD and DIARETDB1	Se = 80.49% Sp = 99.68% Acc = 98.68% DSC = 86.51% IoU = 76.61% Pr = 99.98%
Godlin Atlas et al.^58^	standard scalar, removing of missing values, min-max scalar, DPFE	splat segmentation ELSTM-CNN	DIARETDB2	Se = 98.67% Sp = 98.91% Acc = 99%

**Table 7:** Summary of neovascularization detection using DL techniques

Author	Preprocessing	Methods	Dataset	Performance
Tang et al.^59^	green channel extraction, CLAHE	semantic segmentation CNN	proprietary	Se = 87.72% Sp = 99.76% Pr = 86.96% Jaccard = 76.43% DSC = 84.66%
Tang et al.^60^	green channel extraction, CLAHE, patching, image normalization, data augmentation	Resnet18 and GoogLeNet ensemble classifier	a mix of images from MESSIDOR, DIARETDB0	Acc = 91.57% Se = 85.69% Sp = 97.44% Pr = 97.10%

The limited number of studies in this area highlights the need for more comprehensive datasets and dedicated modeling strategies, as the variability and structural complexity of these lesions continue to challenge robust detection across diverse imaging conditions.

DL approaches for multiple lesion detection in CFIs have evolved toward unified and multi-task architectures capable of simultaneously identifying diverse lesion types, including MAs, HEs, EXs, NV, and SEs. Early studies explored probabilistic and weakly supervised formulations, modeling lesion detection through background representation and coarse-to-fine segmentation strategies.^61^^,^^62^

Recent developments have been driven by encoder-decoder architectures and transformer-based designs that enhance feature representation across lesion types. Variants of U-Net and U-Net++ that incorporate residual connections, multi-branch structures, and multi-scale feature fusion have been widely adopted to address lesion variability.^63^^,^^64^^,^^65^^,^^66^ These approaches often integrate mechanisms such as atrous spatial pyramid pooling and progressive feature aggregation to improve segmentation consistency.

In parallel, detection-oriented frameworks have gained relevance for joint localization and classification. Architectures based on Mask R-CNN and YOLO have been applied to enable real-time multi-lesion detection, leveraging feature pyramid networks and deep backbones for improved spatial representation.^67^^,^^68^^,^^69^ End-to-end segmentation models incorporating class-imbalance-aware loss functions and multi-scale fusion have also been proposed to enhance performance across lesion categories.^70^

Additional strategies have focused on improving feature integration and model robustness using attention mechanisms, optimization techniques, and hybrid learning schemes. These include feature reassembly networks, metaheuristic optimization during training, interpretable probabilistic frameworks, and hierarchical feature aggregation models.^71^^,^^72^^,^^73^

Hybrid and task-specific approaches further combine multi-task learning, adversarial training, and the integration of handcrafted features to refine the detection performance across lesion types.^74^^,^^75^^,^^76^^,^^77^^,^^78^^,^^79^^,^^80^^,^^81^ Table 8 summarizes representative studies on multiple lesion detection using DL techniques.

**Table 8:** Summary of multiple-lesion detection using DL techniques

Author	Lesion	Preprocessing	Methods	Dataset	Performance
Wang et al.^61^	EX, HE, MA, SE	color normalization and blood vessel removal.	weakly supervised method using MoG	Kaggle MESSIDOR	AUC = 99.07%, mAP = 83.94% AUC = 83.94%, mAP = 90.91%
Huang et al.^63^	EX, HE, MA, SE	resizing, cropping, data augmentation, CLAHE	U-Net (DenseNet-161 backbone)	IDRiD	AUC_PR = 86.59%, 65.70%, 42.79%, 59.68%; AUC_ROC = 99.33%, 95.34%, 98.79%, 94.58% (EX, HE, MA, SE)
Yuan et al.^74^	EX, HE, MA, SE	removing black background, CLAHE, Gaussian filtering, data augmentation	GAU-Net, U-Net with self-attention mechanism, PatchGAN classifier	IDRiD	DSC = 75.70%, 76.53%, 50.06%; Pr = 96.10%, 99.63%, 93.30%, 99.84%; Se = 69.02%, Sp = 99.99% (EX, SE, MA, HE)
Santos et al.^67^	EX, HE, MA, SE	elimination of the black background, thresholding, Sobel filtering, sub-blocking, data augmentation	mask R-CNN (ResNeXt-101), region proposal network (RPN)	DDR	Detection: AP = 25.15%, 15.48%, 10.42%, 15.77%; mAP = 16.70%; Segmentation: AP = 26.87%, 15.89%, 12.74%, 13.88%; mAP = 17.35% (EX, SE, MA, HE)
Upadhyay et al.^76^	MA, HE	wavelet-based vessel removal, green channel extraction, CLAHE	U-Net	IDRiD DDR	mAUPR = 65.60% mAUPR = 25.50%
Yin et al.^65^	EX, HE, MA, SE	histogram equalization, CLAHE, Gaussian filter, RGB to LAB color space, data augmentation	Dual-branch U-Net	IDRiD DDR	AUPR = 52.54%, 66.09%, 88.78%, 72.97% AUPR = 19.18%, 49.52%, 58.44%, 23.45% (MA, HE, EX, SE)
Jiang and Zhao^64^	EX, HE, MA, SE	cropping, resizing, data augmentation	U-Net++, ResNet50, AFMA	IDRiD DDR	*m*-DSC = 63.29%, m-IoU = 47.58%, *m*-DSC = 46.07% m-IoU = 30.46%
Sahoo et al.^81^	HE, MA	green channel extraction, median filtering, CLAHE, contrast stretching	adaptive thresholding, global thresholding, feature extraction, super-learning ensemble classifier	DI-ARETDB0; DIARETDB1	Se = 93.55%, 95.29%; Sp = 96.99%, 97.42%; Acc = 96.10%, 96.91% (MA, HE)
Guo et al.^70^	EX, HE, MA, SE	N/A	L-Seg based on VGG16	IDRiD E-ophtha DDR	AUC = 79.45%, 63.74%, 46.27%, 71.13% AUC = 55.46%, 35.86%, 10.52%, 26.48% AUC = 41.71% (EX), 16.87% (EX, HE, MA, SE)
Alyoubi et al.^69^	EX, HE, MA, SE	enhance luminosity, CLAHE, Gaussian filter, cropping, color normalization, data augmentation	YOLOv3	DDR	mAP = 21.60%
Ding et al.^71^	EX, HE, MA, SE	N/A	CNN model based on improved SFLA (SFCNN) classifier	DRIVE; DIARETDB1	Acc = 95%, 88.70%, 87%, 93.70%; Se = 96.90%, 88.20%, 88.90%, 94.50% (EX, HE, MA, SE)
Li et al.^62^	EX, HE, MA, SE	CLAHE, gamma correction, contrast enhancement, intensity range adjustment	dual thresholds, RAU-Net	Local dataset (BTH)	AUC = 93.21%, 80.18%, 84.79%, 61.76% (EX, HE, SE, MA)
Pereira et al.^68^	EX, HE, MA, SE	cropping, SAHI, data augmentation	YOLO-CSP	DDR IDRiD	AP = 37.60%, 36.20%, 23.40%, 31.50%; mAP = 32.18% AP = 51.10%, 43.60%, 35.70%, 36.40%; mAP = 41.70% (EX, SE, MA, HE)
He et al.^66^	EX, HE, MA, SE	data augmentation (horizontal flips, vertical flips, random rescales)	PMCNet	IDRiD DDR E-ophtha	mAUC = 68.08%, DSC = 56.02%, IoU = 43.12% mAUC = 36.44%, DSC = 39.31%, IoU = 32.29% mAUC = 40.90%, DSC = 45.43%, IoU = 30.70%
Kou et al.^78^	EX, MA	green channel extraction, CLAHE	ERU-Net	E-Ophtha IDRiD DDR	AUC = 99.56%, 99.62% AUC = 98.01%, 98.66% AUC = 96.79%, 96.09% (MA, EX)
Playout et al.^75^	Red and bright lesions	illumination equalization, Gaussian filter, CLAHE, data augmentation (rotation, shearing, elastic distortion, horizontal flipping, scaling, gamma, brightness, saturation, contrast)	convolutional multi-task architecture with weakly supervised learning	DIARETDB1	Bright lesions: Se = 88.29%, Sp = 99.93%, Pr = 81.70%, Acc = 99.89%, F1-score = 84.87%; Red lesions: Se = 85.18%, Sp = 99.89%, Pr = 78.96%, Acc = 99.93%, F1-score = 81.95%
Kumar et al.^79^	EX, MA	resizing, scaling	U-Net and autoencoder with channel-wise spatial attention	IDRiD	Acc = 99.94%, Pr = 98.47%, Se = 98.36%
Kundu et al.^77^	Red lesions (HE, MA)	sub-patching, CLAHE	nested U-Net, ResNet-18 classifier	DIARETDB1	Se = 88.79%, Sp = 99.64%, Acc = 99.53%, Pr = 71.50%, F1-score = 79.21%
Tan et al.^72^	EX, HE, MA, SE	Gaussian filtering, data augmentation	DS-U-Net	IDRiD DDR	mAUC = 70% mAUC = 48.3%
Zhou and Zhang^73^	EX, HE, MA, SE	resizing, data augmentation	RDCMM, MSPM	IDRiD DDR	AUC = 75.66% AUC = 32.86%

These developments reflect a shift toward integrated frameworks capable of modeling both lesion-specific characteristics and spatial relationships. However, the simultaneous representation of multiple lesion types introduces additional complexity, particularly in balancing inter-class interactions and ensuring consistent performance across heterogeneous datasets, which remains an open challenge for multi-lesion modeling.

### Diabetic retinopathy classification with deep learning

DL approaches have become the dominant paradigm for DR detection and classification, enabling end-to-end learning of discriminative features directly from CFIs. Unlike traditional methods based on handcrafted features, deep models integrate feature extraction and classification within a unified framework. Two main strategies have been explored: image-based approaches, which analyze the entire fundus image to predict DR severity, and lesion-based approaches, which explicitly incorporate pathological structures into the classification process. The following subsections summarize these paradigms, with emphasis on architectural design, methodological trends, explainability, and emerging directions in DR analysis.

#### Image-based method

Image-based approaches treat the entire fundus image as an input to DL models, typically leveraging transfer learning from pre-trained architectures. A wide range of CNN backbones has been explored for DR classification in different experimental settings. Combinations of pre-trained models have been evaluated to improve the classification performance, while other studies have focused on EfficientNet- and ResNet-based architectures for severity classification.^82^^,^^83^^,^^84^^,^^85^^,^^86^ Variants of lightweight and modified CNNs have also been investigated for both detection and classification tasks.^87^^,^^88^^,^^89^

Model comparison and transfer learning strategies have received significant attention, with multiple studies evaluating different architectures and training configurations, including fine-tuning and feature extraction schemes.^90^^,^^91^^,^^92^^,^^93^ In parallel, attention mechanisms and feature enhancement techniques have been introduced to improve spatial representation and address class imbalances.^94^^,^^95^

To improve robustness, ensemble and hybrid approaches have been proposed, which combine multiple CNN architectures or integrate recurrent components, such as LSTM, to capture complex feature dependencies.^96^^,^^97^^,^^98^^,^^99^

A broad range of architectures, including DenseNet, Xception, ResNet, EfficientNet, and inception-based models, have been explored across diverse datasets and training configurations, with studies focusing on the impact of architectural design and transfer learning on classification performance.^100^^,^^101^^,^^102^^,^^103^^,^^104^^,^^105^^,^^106^^,^^107^^,^^108^^,^^109^^,^^110^^,^^111^^,^^112^^,^^113^^,^^114^

Optimization-based strategies have also been investigated to enhance parameter tuning and model performance, incorporating metaheuristic algorithms such as PSO, GA, ACO, and HHO into DL frameworks.^115^^,^^116^^,^^117^^,^^118^^,^^119^^,^^120^^,^^121^^,^^122^^,^^123^^,^^124^^,^^125^^,^^126^ Additional approaches have combined cross-scale attention mechanisms and hybrid learning schemes, integrating deep feature extraction with classical classifiers.^127^^,^^128^

Image-based approaches provide a scalable and efficient framework for DR classification, benefiting from end-to-end learning and the ability to leverage large, annotated datasets without requiring explicit lesion-level annotations. However, their reliance on global image representations limits interpretability and reduces transparency in clinical decision-making because predictions are not directly linked to specific pathological features. This limitation has motivated the development of lesion-based approaches that aim to incorporate clinically meaningful structures into the classification process.

#### Lesion-based method

Lesion-based approaches aim to support DR classification by explicitly detecting and analyzing pathological structures, such as MAs, HEs, EXs, and SEs. In contrast to image-based models, these methods seek to emulate clinical reasoning by incorporating the lesion type, distribution, and severity into the decision-making process, providing improved interpretability.

Early and hybrid frameworks combined classical image processing with DL models, integrating preprocessing, saliency detection, morphological operations, and handcrafted feature extraction with CNN-based classification.^129^^,^^130^^,^^131^^,^^132^ Optimization-based strategies and patch-level analyses have also been explored to enhance feature discrimination and lesion localization.^133^^,^^134^

Lesion-aware architectures that integrate segmentation and classification within a unified framework have gained prominence. These include attention-based models designed to capture dependencies between lesion types, as well as multi-branch and multi-stage architectures that combine segmentation, classification, and multi-scale feature fusion.^135^^,^^136^^,^^137^^,^^138^^,^^139^

Detection and localization strategies have further improved clinical alignment by explicitly identifying lesion regions prior to classification, often combining global image analysis with object detection frameworks or sequential modeling techniques.^69^^,^^140^^,^^141^

Additional hybrid approaches have incorporated optimization techniques, ensemble learning, and multi-modal or sequence-based architectures to refine performance and capture temporal or contextual dependencies.^142^^,^^143^^,^^144^^,^^145^^,^^146^^,^^147^ Table 9 summarizes the representative lesion-based DR detection methods.

**Table 9:** Summary of lesion-based DR detection studies

Authors	Methods	Dataset	Performance
Sudha and Ganeshbabu^129^	saliency detection, structure tensor, active contour approximation, estimation of ratio, feature extraction with K-means, VGG19 classification	Kaggle	Acc = 98.92%, Pr = 93.90%, Sp = 98.95%, Se = 93.3%
Liu and Chi^135^	cross-lesion attention network (CLANet), cross-scale context attention (CSCA) module, multi-scale context feature fusion, fully connected layer classifier	IDRiD MESSIDOR DDR	Acc = 71.84%, Kappa = 80.12% Acc = 78.17%, Kappa = 87.55% Acc = 79.12%, Kappa = 80.84%
Jabbar et al.^133^	image resizing, green channel extraction, top hat, bottom hat, data augmentation, APSO with GoogleNet and ResNet, classifiers: RF, SVM, DT, NB	Kaggle	Acc = 94%, Pr = 97%, Se = 89%, F1-score = 96%
Abdelmaksoud et al.^130^	preprocessing (median filter, HEBPDS, green channel extraction), GLRM, SVM bi-class classifier, U-Net segmentation, feature extraction, SVM multi-class	HRF; ChaseDB1; DIARETDB0; DIARETDB1; STARE; MESSIDOR; DRIVE; IDRiD	Acc = 95.1%, AUC = 91.9%, Se = 86.1%, Sp = 86.8%, DSC = 86.2% (average over datasets)
Hassan et al.^136^	preprocessing (CLAHE, patch creation, data augmentation), feature extraction with VGG16 and ResNet50, lesion segmentation with U-Net, XGBoost for DR detection	MESSIDOR-2 IDRiD	Acc = 100%, Pr = 100%, Se = 100%, F1-score = 100%, AUC = 100%
Saranya et al.^132^	preprocessing (resizing, binarization, CLAHE, morphological operations), U-Net (red lesion detection), CNN classification	MESSIDOR	Sp = 93.80%, Se = 92.30%, Acc = 95.94%
Devaraj et al.^131^	preprocessing (green channel extraction, optic disc masking, top and bottom hat, median filtering, contrast stretching), local entropy thresholding (LET), feature extraction, NN classifier	DIARETDB0; DIARETDB1; proprietary dataset	Acc = 93.90%, Pr = 92.50%
Amalia et al.^146^	preprocessing (cropping, CLAHE), CNN for lesion features, RNN for DR detection (AlexNet, VGGNet, GoogleNet, LSTM)	MESSIDOR	Acc = 96.12% (GoogleNet)
Zago et al.^134^	patching, VGG16 for patch selection, probability maps	MESSIDOR Kaggle IDRiD DDR DIARETDB0	AUC = 91.20%, Se = 94% AUC = 76.40%, Se = 91.10% AUC = 81.80%, Se = 84.10% AUC = 84.80%, Se = 89.10% AUC = 78.60%, Se = 82.10%
Alyoubi et al.^69^	preprocessing (CLAHE, Gaussian filter), CNN512, CNN299, YOLOv3	DDR	Acc = 89%, Sp = 97.30%, AUC = 97%
Chen et al.^137^	SR-Net (ResNet50 baseline) with SE-Block attention, MT-SNet (VGG26 and U-Net inspired), SRVGG	DDR	Acc = 90.75%, Se = 91.77%, Sp = 93.96%
Deshmukh et al.^142^	preprocessing (green channel extraction, Gaussian filtering), U-Net segmentation, feature extraction, CNN feature selection, SFATO	IDRiD DDR	Acc = 90.25%, Se = 91.42%, Pr = 89.58% Acc = 91.42%, Se = 92.54%, Pr = 90.54%
Li et al.^138^	preprocessing (resizing, CLAHE, normalization), MSGDA-Net, SFB, ESAB	VisionDR APTOS DDR IDRiD	Acc = 75.00%, Pr = 76.37%, Se = 74.92% Acc = 87.18%, Pr = 77.40%, Se = 74.23% Acc = 75.41%, Pr = 65.01%, Se = 70.86% Acc = 84.38%, Pr = 77.88%, Se = 79.84%
Xia et al.^139^	MPAG (patch lesion attention), LLM (lesion location), global network (classification)	DDR	Acc = 80.64%
Mundada and Nawgaje^140^	Preprocessing (Laplacian filter, ROI extraction, augmentation), U-Net_GSCA, GSCA_DeepCNN	IDRiD	Acc = 92.20%, Se = 92.50%, Sp = 91.90%
Khaparde et al.^141^	preprocessing (discard blurry images, scaling, Laplacian filter), Swin-U-Net, 3D-CNN, DA-RNN	DIARETDB1 RIGA	Acc = 96.62%, Se = 96.62%, Sp = 96.62%, Pr = 90.52% Acc = 96.34%, Se = 96.62%, Sp = 96.25%, Pr = 89.58%
Hariobulesu and Shaik^147^	preprocessing (bilinear interpolation, CLAHE), U-Net, LTCN, SVM	MESSIDOR-2	Acc = 98.83%, Se = 99.21%, Sp = 98.87%, Pr = 99.28%, F1-score = 99.15%
Hemanth et al.^143^	preprocessing (Gaussian filtering, HWBLSTM), EGORGA, SqueezeNet, MSVD, LSTM	APTOS MESSIDOR	Acc = 99.68% Acc = 99.75%
Mishra et al.^144^	preprocessing (grayscale conversion, resizing, adaptive mean thresholding), CNN (4 layers), SVM	EYEPACS	Acc = 94%, Pr = 96%, Se = 91%, F1-score = 93%, AUC = 92%
Srividya and Joshitha^145^	preprocessing (adaptive median filter), EAD-Net, Shepard CNN, LSTM	IDRiD	Acc = 91.9%, Se = 91.2%

These methods provide improved interpretability by linking predictions to clinically meaningful structures; however, their performance is strongly dependent on the Acc of the lesion detection and segmentation stages. Errors in early lesion localization can propagate through the pipeline, affecting classification reliability and limiting robustness in complex clinical scenarios. This highlights the need for tightly integrated frameworks that can jointly model the lesion representation and disease severity.

#### Explainable artificial intelligence in the detection and classification of diabetic retinopathy

The rapid adoption of DL for DR analysis has heightened concerns regarding model interpretability, as high-performing systems often function as black boxes, limiting their clinical applicability.^148^ Explainable AI (XAI) addresses this limitation by providing mechanisms to interpret model decisions and link them to clinically meaningful retinal features.

Post-hoc visualization techniques, particularly Grad-CAM and related saliency-based methods, are widely used to generate class-discriminative heatmaps that highlight regions associated with DR lesions.^149^^,^^150^ On the other hand, attention-based architecture incorporates interpretability directly into the learning process by guiding the model toward relevant spatial and channel-wise features, thereby improving lesion representation. Recently, alternative strategies such as multiple-instance learning (MIL) have enabled intrinsic interpretability by assigning attention weights to image patches, thereby facilitating lesion localization.^151^

Beyond spatial explanations, feature attribution methods, such as Shapley additive explanations (SHAPs), offer complementary insights by quantifying the contributions of image regions or clinical variables to model predictions.^152^^,^^153^^,^^154^ These approaches have demonstrated the ability to identify clinically relevant markers associated with DR. They have also been extended to incorporate systemic risk factors, enabling a more comprehensive interpretation of the disease severity.^155^

Despite these advances, current XAI methods have several limitations. Visual explanations, such as saliency maps and attention heatmaps, do not necessarily reflect causal relationships, and their clinical validity remains difficult to evaluate. Moreover, the lack of standardized evaluation protocols for explanation quality limits reproducibility and comparability across studies in this area. These challenges highlight the need for more rigorous validation frameworks that ensure that explanations are not only visually plausible but also clinically meaningful and consistent across datasets.

#### Emerging paradigms in diabetic retinopathy analysis

Recent advances in DR analysis have been largely driven by the convergence of three major paradigms: transformer-based architectures, self-supervised learning (SSL), and foundation models. These approaches have emerged to overcome important limitations of CNNs, particularly their restricted receptive fields, heavy dependence on large, annotated datasets, and limited generalization across different domains.

Vision transformers (ViTs) and their variants have demonstrated improved performance in DR classification by modeling interactions among spatially distant retinal features, which are often critical for accurate classification.^156^^,^^157^ More advanced designs integrate hierarchical and multi-scale representations, such as Swin-based and multi-branch transformer frameworks.^158^^,^^159^ In addition, hybrid architectures that combine convolutional and transformer components have been proposed to balance local feature Se with global attention.^160^ Recent work has further explored this direction through hybrid CNN-transformer models that integrate complementary feature representations while enhancing interpretability via Grad-CAM.^161^ To address the high-resolution nature of fundus images, recent approaches have incorporated MIL, allowing patch-based processing while preserving global relationships.^162^ Despite these advantages, transformer-based methods typically require large-scale pretraining and remain computationally demanding, limiting their applicability in resource-constrained clinical settings.

SSL has emerged as a powerful approach to address the persistent shortage of annotated medical datasets. Early contrastive learning methods already showed notable improvements in scenarios with limited labeled data.^163^ More recently, researchers have incorporated transformer-based backbones and saliency-guided strategies to better capture clinically relevant regions.^164^ Advanced techniques such as masked autoencoders (MAEs) and anatomy-aware masking strategies have further enhanced the modeling of both local and global features, as well as subtle lesion patterns.^165^ Hybrid SSL frameworks combining CNNs, attention mechanisms, and uncertainty-aware learning have also been proposed to increase robustness and better support clinical decision-making.^166^ Other works have explored equivariant learning and multi-instance representations to improve lesion localization and classification.^167^^,^^168^

Overall, SSL significantly reduces dependence on large, labeled datasets and improves generalization. However, its success still heavily depends on the quality and diversity of the data used during pretraining.

Foundation models have introduced a significant paradigm shift in ophthalmic imaging, enabling more generalizable and label-efficient learning across various tasks. For instance, large-scale models such as RETFound have shown strong transferability to different retinal applications.^169^ Moreover, multimodal foundation models combined with in-context learning strategies now enable rapid adaptation to new tasks with little or no additional retraining, often achieving performance levels comparable to specialized models for DR detection.^170^ Some studies have also explored integrating foundation models with domain-specific knowledge, such as gaze-guided prompting and human-in-the-loop approaches, demonstrating promising improvements in the detection and annotation of small lesions within clinical workflows.^171^

However, several challenges persist across emerging paradigms. Transformer-based and foundation models often require large amounts of computational resources and data for effective deployment, whereas SSL approaches may still struggle to capture rare pathological patterns without sufficient domain diversity. Cross-dataset generalization remains a persistent issue, particularly when models are trained on homogeneous datasets. These limitations suggest that future research should focus on integrating the strengths of these paradigms by combining global modeling, data-efficient learning, and scalable pretraining while improving robustness, interpretability, and clinical applicability.

## Discussion

### Dataset challenges and generalization

A wide variety of publicly available datasets have been used for both lesion detection and DR classification; however, most of them are relatively small, except for the EYEPACS dataset, which contains 88,702 images. In the present review, 95,674 images were analyzed, of which approximately 93% corresponded to the EYEPACS. This imbalance highlights a strong dependence on a limited number of large-scale datasets, whereas smaller datasets, such as IDRiD, DDR, E-ophtha, and DIARETDB1, remain widely used for lesion-level tasks. Similarly, DR detection and classification studies predominantly rely on the IDRiD, DDR, MESSIDOR, and Kaggle/APTOS2019 datasets. These observations suggest that although multiple datasets are available, the field remains constrained by the dataset size, variability, and annotation availability.

In addition, several dataset-related challenges must be considered when interpreting the reported performance. Class imbalance is common, particularly in multi-class DR classification, where advanced stages are underrepresented relative to no-DR or mild cases, potentially inflating the overall Acc while reducing the Se of clinically relevant classes. Variations in image quality, illumination, and acquisition conditions may further affect model stability, motivating the development of preprocessing and enhancement strategies to improve retinal image consistency across datasets.^172^ Label noise is also a significant limitation because DR classification depends on expert interpretation and may be affected by inter-observer variability, image quality, and differences in annotation protocols. Furthermore, inter-dataset variability and domain shift, arising from differences in acquisition devices, population characteristics, and preprocessing pipelines, can significantly affect model generalization, meaning that high performance on a single dataset may not translate to real-world clinical settings.^173^^,^^174^^,^^175^^,^^176^

### Methodological paradigms for DR analysis

Regarding lesion detection, two main methodological paradigms have been identified: traditional non-DL approaches based on handcrafted features and image-processing techniques and DL-based approaches that rely on CNNs and related architectures. Although traditional methods provide interpretable pipelines, their performance is highly dependent on feature engineering and preprocessing. In contrast, DL-based methods enable end-to-end learning and have become the dominant approach, particularly because of their ability to capture complex feature representations; however, this comes at the cost of increased computational requirements and dependence on large, annotated datasets.

In lesion-specific analyses, most studies focused on multiple-lesion detection (49%), followed by MAs (22%) and exudates (19%), whereas HEs and NV remain comparatively underexplored. This imbalance likely reflects current gaps in the literature rather than a selection bias in this review and may be partially explained by the clinical emphasis placed on certain lesion types and the technical challenges associated with detecting small, sparse, or less frequently annotated abnormalities.

Two main strategies were identified for DR classification: image-based and lesion-based approaches. Image-based methods, which account for approximately 73% of the reviewed studies, process the entire fundus image and leverage global contextual information for classification. These approaches are widely adopted because of their simplicity, scalability, and strong performance. However, they often lack interpretability because they do not explicitly identify the lesions contributing to the classification decisions.

In contrast, lesion-based approaches (27%) aim to mimic clinical reasoning by detecting and analyzing specific pathological structures before classification. These methods offer improved interpretability and closer alignment with ophthalmological practice but are more complex and depend heavily on accurate lesion segmentation. In addition, their relatively limited adoption indicates that further research is required to fully assess their robustness and clinical applicability.

**Figure 9:**
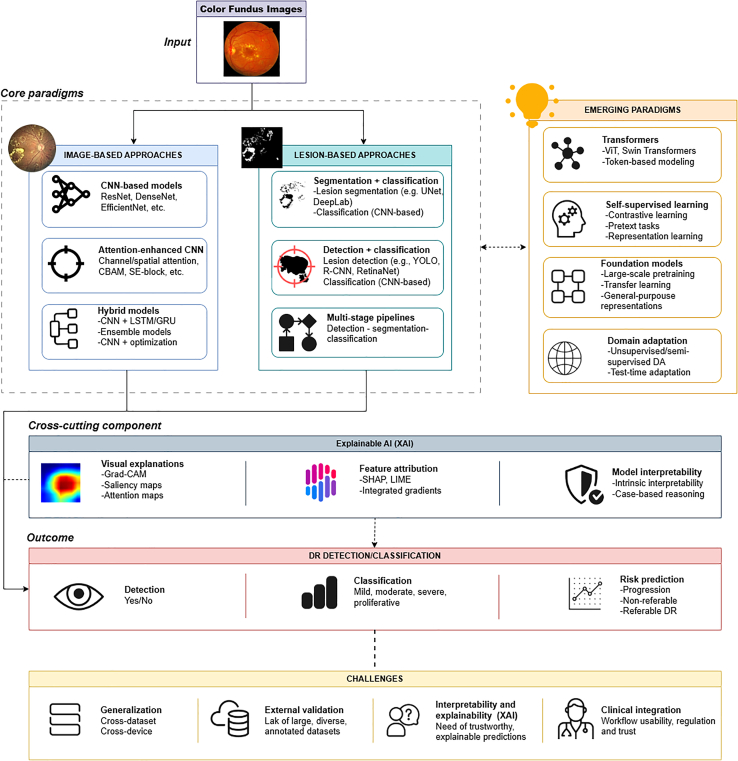
Conceptual taxonomy and unified framework of deep learning approaches for diabetic retinopathy analysis in color fundus images

The taxonomy in Figure 9 organizes DL approaches for DR analysis into two core paradigms: image-based methods, which leverage global fundus features for DR detection and classification, and lesion-based methods, which explicitly identify and quantify pathological structures to support clinical interpretation. Emerging paradigms have been highlighted as extensions that enhance feature representation and robustness across the datasets. XAI is presented as a cross-cutting component that emphasizes visual explanations, feature attribution, and model interpretability to bridge the gap between automated predictions and clinical trust. This framework provides a structured view of the field, supporting the comparative analysis and discussion of the challenges presented in the subsequent sections.

A comparative analysis of the DL paradigms, summarized in Table 10, highlights the distinct trade-offs that shape their suitability for DR analysis. CNN-based models effectively capture local lesion patterns and perform robustly with moderate data availability; however, their limited receptive field constrains global context modeling. Transformer-based architectures address this limitation by modeling long-range dependencies; however, they typically require extensive pretraining and substantial computational resources. Hybrid CNN-transformer models partially overcome these limitations by combining local and global feature representations, yielding promising performance across heterogeneous lesion types. Recently, self-supervised and foundation models have enabled data-efficient learning and improved transferability, although their clinical validation remains limited. Overall, no single paradigm is universally optimal, and model selection should be guided by data availability, computational constraints, and the need for generalization in real-world settings.

**Table 10:** Comparative analysis of DL paradigms for DR analysis

Paradigm	Representative approaches (from this review)	Architectural complexity	Data dependency	Strengths	Limitations	Generalization
CNN-based models	ResNet, EfficientNet, DenseNet, U-Net variants	moderate	moderate-high	strong local feature extraction; robust for lesion-level patterns; well-established	limited global context; reduced interpretability in image-based models	moderate; sensitive to dataset bias
Vision transformers	ViT, Swin transformer, multi-branch transformer models	high	high	capture long-range dependencies; improved global context modeling	high computational cost; require large-scale pretraining	high when pre-trained; unstable on small datasets
Hybrid CNN-transformer	CNN-ViT, multi-branch fusion architectures	moderate-high	moderate-high	combine local and global features; improved performance in heterogeneous lesions	increased architectural complexity; training instability	promising across datasets
Self-supervised/foundation models	SSL frameworks, RETFound, multi-modal models	high	high (pretraining stage)	reduce reliance on labeled data; strong transfer learning capability	dependence on large unlabeled datasets; limited clinical validation	high cross-domain potential

Beyond the methodological diversity across studies, a key conceptual trend is the shift from handcrafted, single-model pipelines to integrated, data-driven architectures that capture both global and lesion-level information. Although CNN-based models remain dominant owing to their resilience and scalability, emerging architectures that incorporate attention mechanisms, hybrid learning strategies, and transformer-based components signal a transition toward more context-aware and adaptable systems. From a clinical perspective, image-based approaches offer strong performance and scalability for large-scale screening, whereas lesion-based methods provide improved interpretability and closer alignment with ophthalmology reasoning. However, the limited standardization of evaluation protocols and variability across datasets continue to hinder clinical translation, underscoring the need for more unified benchmarks and clinically validated frameworks.

### Benchmark performance and comparative analysis

Although a formal meta-analysis was not performed owing to the heterogeneity among the included studies, the consolidated comparison in Table 11 provides an overview of the performance trends across commonly used benchmark datasets. To improve readability, AUC values are reported as percentages while preserving the original metrics used in each study. Because the reviewed studies employed heterogeneous evaluation protocols, the primary metric emphasized by each study (typically AUC or Acc) was reported rather than imposing uniformity, which could introduce bias. Overall, studies evaluated on large-scale datasets, such as EyePACS and APTOS, tend to report higher performance, reflecting the benefits of data availability and diversity. In contrast, the results on smaller or more specialized datasets, such as IDRiD and DDR, showed greater variability, highlighting the influence of dataset size, class imbalance, and annotation differences. While AUC provides a more robust measure of discrimination in imbalanced settings, the coexistence of metrics across studies underscores the current lack of standardized evaluation practices in DR research, limiting direct comparability and reinforcing the need for more consistent benchmarks.

**Table 11:** Consolidated performance comparison across benchmark datasets for DR classification

Dataset	Study	Approach Type	Approach	Metric	Value
EyePACS	Suo et al. ^94^	image-based	attention CNN (CS-ResNet-101)	accuracy	98.10%
EyePACS	Sushith et al. ^99^	hybrid	CNN + LSTM + attention	accuracy	96.90%
EyePACS	Mutawa et al. ^84^	image-based	transfer learning (DenseNet121)	accuracy	89.10%
EyePACS	Mishra et al. ^144^	image-based	CNN	AUC	92.00%
APTOS 2019	Mutawa et al. ^84^	image-based	transfer learning (DenseNet121)	accuracy	98.50%
APTOS 2019	Naveen et al. ^128^	hybrid	EfficientNet + SVM	AUC	97.00%
APTOS 2019	Kallel and Echtioui ^92^	image-based	transfer learning (InceptionV3)	accuracy	96.88%
APTOS 2019	Liu et al. ^93^	image-based	transfer learning (InceptionV3)	AUC	92.56%
Messidor	Saranya et al. ^132^	lesion-based	U-Net + CNN	accuracy	95.94%
Messidor	Amalia et al. ^146^	hybrid	CNN + RNN	accuracy	96.12%
Messidor-2	Abbasi et al. ^96^	image-based	DCNN	AUC	98.23%
Messidor-2	Hariobulesu and Shaik ^147^	hybrid	U-Net + SVM	accuracy	98.83%
IDRiD	Mundada and Nawgaje ^140^	lesion-based	U-Net + CNN	accuracy	92.20%
IDRiD	Abdelmaksoud et al. ^130^	lesion-based	U-Net + SVM	AUC	91.90%
DDR	Chen et al. ^137^	image-based	Attention CNN	accuracy	90.75%
DDR	Alyoubi et al. ^69^	hybrid	CNN + YOLO	AUC	97.00%

A critical limitation of the reviewed studies is the limited evaluation of model generalization in cross-dataset settings. Most approaches report performance on a single dataset or randomly split subsets, which may not reflect the real-world variability in imaging conditions, patient populations, and annotation standards. As shown in Table 11, the performance can vary substantially when the models are evaluated across different datasets, indicating their Se to the distribution shifts. This behavior has been reported in recent studies, where models tend to learn domain-specific characteristics rather than clinically relevant features, leading to performance degradation under domain shift.^177^ Furthermore, variability across publicly available datasets, including differences in image quality, acquisition devices, and classification protocols, has been identified as a key factor limiting model cross-domain performance and transferability.^173^ This lack of external validation raises concerns regarding the robustness and clinical reliability of current DL systems for DR classification. In this context, emerging paradigms such as SSL and foundation models may improve generalizability by leveraging large-scale, diverse datasets, although their evaluation in cross-dataset settings remains limited.

### Emerging directions and clinical translation

This review focuses on CFI to ensure methodological consistency across studies, as well as its widespread use in large-scale DR screening programs. However, recent advances in retinal imaging suggest that multi-modal approaches that combine fundus images with complementary modalities, such as optical coherence tomography (OCT), can provide additional structural and depth-resolved information that is not captured by 2D fundus images alone.^178^^,^^179^ By integrating surface-level and cross-sectional retinal features, these frameworks have demonstrated improved and consistent diagnostic performance, particularly in challenging cases with limited or ambiguous lesion visibility.^180^ Although these approaches fall outside the scope of this review, they highlight a relevant direction for future research, particularly in improving the robustness, cross-dataset reliability, and clinical integration of DR classification systems.

Despite significant advances in DL for DR detection and classification, its clinical applications remain limited. Several practical challenges continue to hinder real-world deployment, including the computational demands of real-time inference, particularly in resource-constrained screening settings and remote healthcare environments, where lightweight and efficient systems are required to support large-scale screening programs.^181^^,^^182^ In addition, regulatory approval processes require robust evidence of safety, reliability, and generalization, which is often lacking in current studies, and regulatory pathways vary across jurisdictions.^183^ Broader discussions on AI governance and national policy frameworks further highlight the importance of regulatory and ethical considerations in integrating AI technologies into healthcare systems.^184^ Integrating models into clinical workflows also poses challenges, as they must be compatible with existing screening programs, support clinician decision-making, and provide interpretable outputs. Real-world implementation studies of DL healthcare applications demonstrate the need for structured clinical evaluation and sustainability frameworks.^185^ Addressing these factors is essential to bridge the gap between high-performance research models and clinically deployable systems.

### Research opportunities and challenges

Future research should focus on addressing several challenges to facilitate the clinical translation of DL systems for DR. First, particular attention should be given to developing lightweight, computationally efficient models suitable for low-resource settings, especially in low-income countries, where real-time inference on mobile or edge devices is essential. Second, improving the interpretability of the model remains a major priority. The adoption of XAI techniques, such as Grad-CAM, SHAP, and attention mechanisms, may strengthen clinician trust and facilitate integration into clinical workflows. Third, fairness and bias mitigation require greater attention. Future studies should evaluate and reduce demographic biases related to ethnicity, sex, and socioeconomic status to ensure equitable performance across diverse populations. Additionally, federated learning approaches should be further explored to enable collaborative model training across institutions while preserving patient data privacy, given the Se of medical imaging data. Finally, the current dataset limitations, including class imbalance, label noise, and domain shift, continue to restrict model robustness and generalization. Addressing these issues through domain adaptation strategies, standardized evaluation protocols, and multi-modal integration, such as combining fundus images with OCT, is essential for improving clinical reliability and real-world applicability.

### Conclusion

DR remains a major cause of vision impairment, and early detection and accurate diagnosis are essential for preventing disease progression. This systematic review provides a comprehensive analysis of recent advances in DL for retinal lesion detection and DR classification using CFI, covering both lesion-level approaches and whole-image-based detection and classification strategies.

The reviewed studies indicate that DL models, particularly CNNs and their variants, have become the predominant approach owing to their strong performance and ability to learn complex feature representations. However, the analysis also revealed variability in evaluation practices, with different combinations of performance metrics used across studies, making direct comparisons challenging.

This review consolidates current methodologies, datasets, and evaluation strategies, offering a structured perspective on lesion detection and DR severity classification. In particular, the inclusion and characterization of lesion-based approaches provide a relevant reference for future research focused on improving interpretability and clinical alignment. In addition, the scientometric analysis presented offers insights into research trends, including publication patterns, geographic distribution, and thematic evolution, while the compilation of publicly available datasets serves as a practical resource for further development in the field.

Although DL-based methods have achieved significant progress, challenges related to data availability, evaluation consistency, interpretability, and generalization remain. Addressing these limitations is essential for advancing the robustness and clinical applicability of automated DR detection systems.

Overall, this review organizes existing work and highlights key methodological and clinical gaps that must be addressed to enable the reliable and scalable deployment of DL systems in real-world ophthalmic practice.

### Limitations of the study

This review has several limitations. The included studies exhibited substantial heterogeneity in datasets, model architectures, and evaluation protocols, limiting direct comparability and precluding quantitative meta-analysis. In addition, performance reporting is often inconsistent and dataset-specific, making it difficult to draw unified conclusions or assess generalization across different studies. Furthermore, most studies rely on evaluations conducted on single datasets, with limited cross-dataset validation, which restricts insights into real-world robustness. Focusing exclusively on color fundus imaging excludes studies using complementary modalities, such as OCT or multi-modal imaging, which may limit the ability of this analysis to capture recent advances that leverage structural and depth-resolved retinal information to enhance diagnostic performance and robustness. Finally, only English-language journal articles published between 2019 and 2025 were considered, which may have introduced selection bias.

## Acknowledgments

This work was made possible through funding from the Ministry of Science, Technology, and Innovation of Colombia (10.13039/100022965Minciencias) under Framework Project No. 0855-937-106-267.

## Author contributions

Conceptualization, J.A.G., L.R.G., O.T.F., M.G., J.S.C., and J.E.G.; methodology, J.A.G. and E.D.L.H.F.; data curation, E.A.; supervision, E.A., L.R.G., O.T.F., E.D.L.H.F., M.G., and J.S.C.; writing – original draft, J.A.G., M.G., and J.E.G.

## Declaration of interests

The authors declare no conflicts of interest.

## STAR★Methods

### Key resources table

**Table:** 

REAGENT or RESOURCE	SOURCE	IDENTIFIER
**Software**		

Zotero	Digital Scholar	https://www.zotero.org/
Rayyan	RAYYAN	https://www.rayyan.ai/

### Method details

#### Study design and protocol

This systematic review was conducted in accordance with the PRISMA 2020 guidelines to ensure transparency and reproducibility of the study selection and reporting. Given the heterogeneity of the included studies in terms of datasets, methodologies, and evaluation metrics, a quantitative meta-analysis was not performed, and the review focused on qualitative synthesis. Additionally, no formal risk-of-bias assessment was conducted because of variability across studies.

#### Data sources and search strategy

Scopus, Web of Science (WoS), and IEEE Xplore were selected as the primary databases because of their extensive coverage of peer-reviewed literature in biomedical engineering and artificial intelligence. The search was conducted between June 2025 and November 2025 and focused on studies published between 2019 and 2025 to capture the recent advances in the field.

Two query strings were used to identify the relevant studies. For lesion detection and segmentation, the following query was applied: (“Microaneurysm” OR “Exudate” OR “Hemorrhage” OR “Neovascularization” OR “Lesion∗”) AND (“Detection” OR “Localization” OR “Segmentation”). For diabetic retinopathy detection and classification, the query used was: (“Diabetic retinopathy” OR “DR”) AND (“Classification” OR “Screening” OR “Grading” OR “Detection”). These queries were adapted to the syntax of each database. These query strings were adapted to the specific syntax requirements, and the records retrieved from Scopus, Web of Science (WoS), and IEEE were imported into the reference management software Zotero and the systematic review platform Rayyan for organization, deduplication, and screening.

#### Study selection process

The study selection process followed the PRISMA 2020 framework. A total of 616 records were initially identified in the selected databases (Scopus: n = 265; Web of Science: n = 136; IEEE Xplore: n = 215). After removing 219 duplicate records, 397 studies remained for screening.

Titles and abstracts were reviewed, and 210 records that did not meet the predefined criteria were excluded from the study. Subsequently, 187 full-text articles were assessed for eligibility, of which 41 were excluded for the use of non-CFI imaging modalities, lack of relevance to DR, or insufficient alignment with the research objectives. Finally, 146 studies were included in the qualitative analysis.

#### Inclusion and exclusion criteria

Studies were included if they were peer-reviewed journal articles published in English between 2019 and 2025 and focused on lesion detection or segmentation related to DR using either non-DL or DL approaches, or on DR detection, or classification using DL techniques. Only studies that employed CFI were considered to ensure modality consistency.

Studies were excluded if they were non-peer-reviewed or secondary literature, including conference papers, reviews, book chapters, editorials, and short surveys, or if they were published before 2019 or not written in English. Studies that did not address DR-related lesion detection or DR classification tasks or relied on alternative imaging modalities, such as Optical Coherence Tomography (OCT) or Indocyanine Green Angiography (ICG), were excluded.

#### Data extraction and synthesis

The following information was extracted from each included study: author, publication year, dataset, methodological approach, evaluation metrics, and main findings. The extracted data were used to support qualitative synthesis and comparative analysis across studies. A qualitative synthesis was conducted to analyze the trends in DL approaches for lesion detection and DR classification. The studies were grouped by methodological paradigm, including non-DL approaches, DL-based lesion detection methods, and image- and lesion-based classification strategies. Owing to the heterogeneity of the datasets, evaluation protocols, and reporting standards, a quantitative meta-analysis was not feasible.

### Quantification and statistical analysis

No formal statistical meta-analysis was conducted due to substantial heterogeneity among the included studies, including differences in datasets, class distributions, evaluation protocols, and reported performance metrics. Instead, the analysis was based on qualitative synthesis, complemented by a structured, descriptive comparison of representative performance metrics across benchmark datasets. This consolidated comparison identifies general performance trends while avoiding potential bias from metric normalization or inappropriate aggregations. Descriptive statistics were also used in the scientometric analysis to summarize the publication trends, geographic distribution, and journal impact.

## Supplemental information

Document S1. PRISMA_2020_checklist
